# A Low T Regulatory Cell Response May Contribute to Both Viral Control and Generalized Immune Activation in HIV Controllers

**DOI:** 10.1371/journal.pone.0015924

**Published:** 2011-01-31

**Authors:** Peter W. Hunt, Alan L. Landay, Elizabeth Sinclair, Jeffrey A. Martinson, Hiroyu Hatano, Brinda Emu, Philip J. Norris, Michael P. Busch, Jeffrey N. Martin, Cicely Brooks, Joseph M. McCune, Steven G. Deeks

**Affiliations:** 1 Departments of Medicine and Laboratory Medicine, University of California San Francisco, San Francisco, California, United States of America; 2 Department of Immunology/Microbiology, Rush University Medical Center, Chicago, Illinois, United States of America; 3 Blood Systems Research Institute, San Francisco, California, United States of America; University of Toronto, Canada

## Abstract

HIV-infected individuals maintaining undetectable viremia in the absence of therapy (HIV controllers) often maintain high HIV-specific T cell responses, which has spurred the development of vaccines eliciting HIV-specific T cell responses. However, controllers also often have abnormally high T cell activation levels, potentially contributing to T cell dysfunction, CD4+ T cell depletion, and non-AIDS morbidity. We hypothesized that a weak T regulatory cell (Treg) response might contribute to the control of viral replication in HIV controllers, but might also contribute to generalized immune activation, contributing to CD4+ T cell loss. To address these hypotheses, we measured frequencies of activated (CD38+ HLA-DR+), regulatory (CD4+CD25+CD127^dim^), HIV-specific, and CMV-specific T cells among HIV controllers and 3 control populations: HIV-infected individuals with treatment-mediated viral suppression (ART-suppressed), untreated HIV-infected “non-controllers” with high levels of viremia, and HIV-uninfected individuals. Despite abnormally high T cell activation levels, controllers had lower Treg frequencies than HIV-uninfected controls (P = 0.014). Supporting the propensity for an unusually low Treg response to viral infection in HIV controllers, we observed unusually high CMV-specific CD4+ T cell frequencies and a strong correlation between HIV-specific CD4+ T cell responses and generalized CD8+ T cell activation levels in HIV controllers (P≤0.001). These data support a model in which low frequencies of Tregs in HIV controllers may contribute to an effective adaptive immune response, but may also contribute to generalized immune activation, potentially contributing to CD4 depletion.

## Introduction

The HIV vaccine field has returned “back to basics” after a T cell-mediated immunity vaccine recently failed to prevent HIV infection and actually increased the risk of infection in important subgroups of individuals [1]. Part of this process is a re-examination of the mechanisms by which some HIV-infected individuals spontaneously control viral replication in the absence of antiretroviral therapy. These HIV controllers represent fewer than 1% of chronically HIV-infected individuals and maintain clinically undetectable plasma HIV RNA levels (operationally defined as <75 copies/ml) in the absence of antiretroviral medications [2], [3], [4], [5]. Several functional immunologic and host genetics studies suggest that high levels of HIV-specific CD4+ and CD8+ T cells with preserved function are likely to play an important role in the suppression of viral replication in most of these individuals [6], [7], [8], [9], [10], [11], [12], [13], [14], [15], [16], [17], [18], [19], observations which have spurred the development of T cell immunity vaccines for HIV. However, the mechanisms of viral control in these individuals are likely to be heterogeneous as many HIV controllers lack a protective HLA type, have very low frequencies of HIV-specific T cells, or maintain control of viral replication even after documented escape from HLA-restricted epitopes [14], [20], [21], [22].

It is important to recognize this heterogeneity as some mechanisms of viral control may prevent both initial infection and clinical progression better than others. For example, high T cell activation and low regulatory T cell (Treg) responses in highly exposed HIV-uninfected individuals have been consistently associated with an increased risk of subsequent HIV infection [23], [24], [25], [26], [27]. Higher T cell activation has also been independently associated with more rapid CD4+ T cell decline and clinical progression to AIDS in untreated HIV-infected individuals [28], [29], [30], [31], [32], [33]. This potentially harmful effect of activation has even been observed among controllers [34]. Persistent immune activation in HIV controllers may also contribute to accelerated atherosclerosis and other non-AIDS morbidities linked to inflammation [35]. Understanding why some mechanisms of viral control are associated with negative inflammatory consequences is therefore an important issue for HIV vaccine development.

We hypothesized that an unusually low Treg response to viral infection might allow some HIV controllers to maintain strong antiviral immune responses at the cost of at the cost of abnormally high generalized immune activation, potentially contributing to CD4+ T cell decline even in the absence of clinically detectable viremia. To address these hypotheses, we measured frequencies of activated (CD38+ HLA-DR+), regulatory (CD4+CD25+CD127^dim^), HIV-specific, and CMV-specific T cells in a large cohort of HIV controllers. We compared these data to those observed in three well characterized control populations: HIV-infected individuals with treatment-mediated viral suppression, untreated HIV-infected “non-controllers” with high levels of viremia, and HIV-uninfected individuals.

## Results

### Characteristics of participants

A total of 52 HIV controllers with plasma HIV RNA levels <75 copies/ml in the absence of antiretroviral therapy, 176 ART-suppressed participants, 72 untreated HIV-infected non-controllers with plasma HIV RNA levels >10,000 copies/ml, and 38 HIV-uninfected participants contributed to these studies. Most were men between 40 and 50 years of age, although compared to other HIV-infected groups, HIV controllers were more likely to be women (P = 0.006, Table 1). The HIV controllers were also much more likely to be hepatitis C virus (HCV) sero-positive than the other HIV-infected groups (70% vs. 38%, P<0.001). While all HIV controllers had plasma HIV RNA levels <75 copies/ml, 19 (37%) had an episode of a clinically measurable plasma HIV RNA level >75 copies/ml in the previous year. While the HIV controllers had significantly higher median CD4+ T cell counts than the ART-suppressed (683 vs. 449 cells/mm^3^, P<0.001) and the non-controllers (683 vs. 251 cells/mm^3^, P<0.001), 9 HIV controllers (17%) had CD4+ T cell counts below 350 cells/mm^3^ and 4 (7%) met the clinical definition of AIDS (one with Kaposi's sarcoma and three with CD4+ T cell counts persistently <200 cells/mm^3^) despite maintaining viral suppression in the absence of therapy.

**Table 1:** Characteristics of Participants Contributing to T Cell Activation and HIV-specific T Cell Response Analyses.

Characteristic	HIV-uninfectedN = 38Median (IQR)	HIV-infectedControllersVL<75 copies/mlN = 52Median (IQR)	HIV-infectedAntiretroviral-treatedVL<75 copies/mlN = 176Median (IQR)	HIV-infectedUntreatedVL>10^4^ copies/mlN = 72Median (IQR)
Age, years	43 (37 to 42)	48 (45 to 52)	46 (41 to 52)	44 (40 to 49)
Female gender, no. (%)	8 (22)	16 (31)	28 (16)	12 (17)
CD4 count, cells/mm^3^	-	683 (466 to 942)	449 (302 to 652)	251 (169 to 395)
Plasma HIV RNA level, log_10_ copies/ml	-	<1.9	<1.9	4.5 (4.2 to 4.9)
Hepatitis C seropositive, no. (%)	-	24 (71)^1^	47 (27)	23 (36)
Duration of HIV Diagnosis, years	-	16 (10 to 19)	13 (8 to 17)	13 (9 to 16)

VL, Plasma HIV RNA Level.

1 Hepatitis C virus serology was unavailable for 18 of 52 controllers.

### HIV controllers have low Treg frequencies despite higher T cell activation

**Figure 1:**
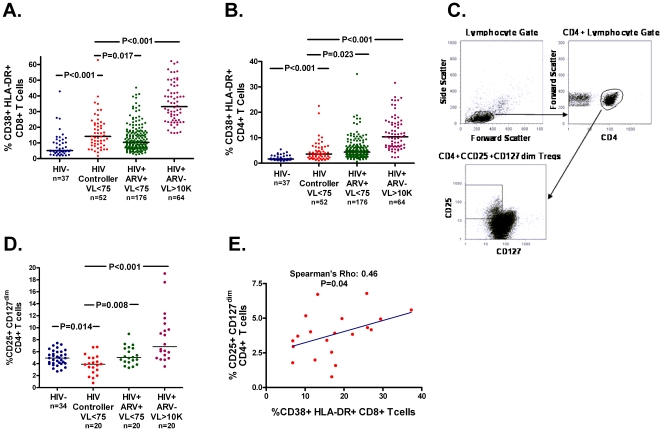
HIV Controllers Have Abnormally Low Treg Frequencies Despite Abnormally High T Cell Activation. The frequency of activated (CD38+ HLA-DR+) CD8+ T cells (**A**) and CD4+ T cells (**B**) in fresh whole blood was compared between 52 HIV-infected untreated HIV controllers, 37 HIV-uninfected participants, 176 HIV-infected participants with plasma HIV RNA levels <75 copies/ml on antiretroviral therapy, and 64 untreated HIV-infected participants with plasma HIV RNA levels >10,000 copies/ml. Cryopreserved PBMC from 34 healthy HIV-uninfected participants in ACTG 5015 (HIV-), 20 HIV controllers, 20 antiretroviral therapy (ART)-treated participants with plasma HIV RNA levels <75 copies/ml and 20 untreated HIV-infected participants with plasma HIV RNA levels >10,000 copies/ml were also evaluated for the frequency of CD4+ Tregs (CD25+CD127^dim^). PBMC preparations were first gated on lymphocytes based on their forward and side scatter properties, then gated for CD4^+^ lymphocytes, then CD4+ lymphocytes positive for CD25 and only dimly expressing CD127, results expressed as a percentage of the parent CD4^+^ population (**C**). HIV controllers had lower frequencies of Tregs than HIV-uninfected controls and both other HIV-infected groups (**D**). Among HIV controllers, higher frequencies of activated CD8+ T cells were associated with higher frequencies of Tregs (**E**). The curve represents the best-fit linear regression model.

We and others have previously reported that most HIV controllers maintain strikingly high frequencies of CD4+ and CD8+ T cells producing interferon (IFN)-γ and interleukin (IL)-2 in response to HIV Gag peptides [14], [19], [22], consistent with their potential role in the control of viral replication. However, as our group has recently reported in a smaller subset of participants (n = 30) [34], HIV controllers also had significantly higher frequencies of activated (CD38+ HLA-DR+) CD8+ T cells (Figure 1A) and CD4+ T cells (Figure 1B) than HIV-uninfected participants (P<0.001 for both), even when restricting to HCV-uninfected individuals (P<0.001). HIV controllers also had higher frequencies of activated CD8+ T cells than ART-suppressed participants (P = 0.017), even after adjustment for HCV sero-status, CD4+ T cell count, and gender (P = 0.056). As we have previously reported [34], higher frequencies of activated CD4+ and CD8+ T cells were associated with greater CD4+ T cell depletion in HIV controllers (P<0.001 for both, Figures S1A and S1B).

We hypothesized that a low Treg response to HIV infection might explain why most HIV controllers maintain high HIV-specific T cell responses but also high generalized T cell activation levels. To assess this possibility, we sampled cryopreserved peripheral blood mononuclear cells (PBMC) from 20 HIV controllers, 20 ART-suppressed, and 20 untreated non-controllers, and 34 healthy HIV–uninfected controls and compared the frequencies of CD25+CD127^dim^ CD4+ Tregs between groups. Despite having higher frequencies of activated CD4+ and CD8+ T cells than HIV-uninfected controls, the HIV controllers had a lower median frequency of Tregs (3.9% vs. 4.9%, P = 0.014, Figure 1C). The HIV controllers also had a lower median frequency of Tregs than the ART-suppressed (3.9% vs. 5.0%, P = 0.008) and non-controllers (3.9% vs. 6.8%, P<0.001). While there was no evidence for a difference in Treg frequencies by gender within either group, among both HIV-uninfected and HIV-infected individuals, lower CD4+ T cell counts were associated with higher frequencies of regulatory T cells (rho: -0.60, P<0.001). To account for differences in absolute CD4+ T cell counts, we compared absolute regulatory T cell counts between groups. While absolute regulatory T cell counts were similar between HIV-infected groups, the HIV controllers continued to have a lower median CD25+CD127^dim^ regulatory CD4+ T cell count than HIV-uninfected participants (33 vs. 40 cells/mm^3^, P = 0.004).

It is surprising that HIV controllers have lower Treg frequencies and counts than HIV-uninfected individuals since higher levels of antigen stimulation and inflammation would be expected to cause greater expansion of Tregs [36]. Supporting this hypothesis, higher plasma HIV RNA levels were strongly associated with higher frequencies of Tregs among HIV-infected non-controllers (rho: 0.72, P<0.001). Furthermore, among HIV controllers, higher frequencies of regulatory T cells were associated with higher frequencies of activated CD4+ T cells (rho: 0.49, P = 0.03) and activated CD8+ T cells (rho: 0.46, P = 0.04, Figure 1D). Based on these latter observations, we would have expected to observe higher Treg frequencies in HIV controllers than in HIV-uninfected individuals as a consequence of greater antigen stimulation and T cell activation. The observation that HIV controllers actually have lower Treg frequencies than HIV-uninfected individuals thus suggests that HIV controllers have an unusually weak Treg response to HIV infection, potentially contributing to the high HIV-specific T cell responses and generalized T cell activation observed.

### Strong relationship between adaptive HIV-specific immune response and generalized T cell activation in HIV controllers

**Figure 2:**
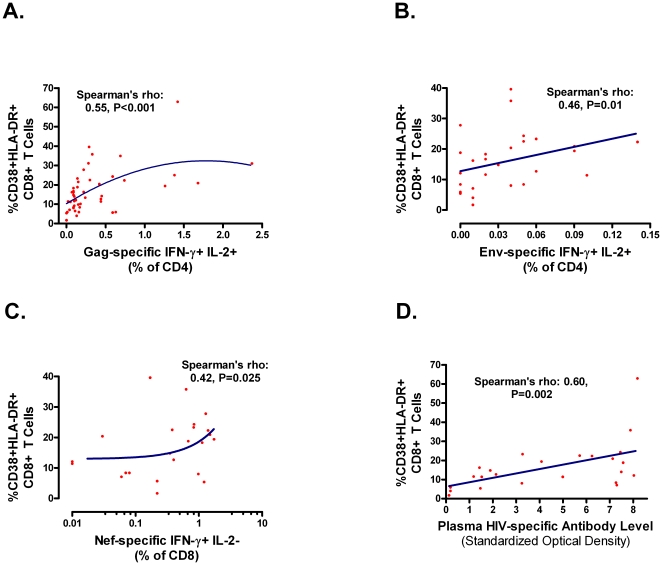
Relationship between Adaptive HIV-specific Immune Responses and CD8+ T Cell Activation in HIV Controllers. The association between the frequency of activated (CD38+ HLA-DR+) CD8+ T cells and the frequency of CD4+ T cells producing both IFN-γ and IL-2 after stimulation with overlapping HIV Gag (**A**) or HIV Env peptides (**B**), CD8+ T cells producing only IFN-γ after stimulation with overlapping Nef peptides (**C**), and plasma HIV-specific antibody levels (as assessed by de-tuned ELISA, **D**) were assessed among HIV Controllers. The curves in each plot represent best-fit linear or quadratic regression models using untransformed data.

Since unusually low Treg responses in HIV controllers might allow for both stronger adaptive HIV-specific immune responses and generalized T cell activation, we hypothesized that there would be a strong relationship between these two latter factors. Among HIV controllers, higher frequencies of CD4+ T cells producing both IFN-γ and IL-2 in response to stimulation with HIV Gag peptides were strongly associated with higher frequencies of activated CD4+ T cells (rho: 0.36, P = 0.012) and activated CD8+ T cells (rho: 0.55, P<0.001, Figure 2A). Higher frequencies of HIV Env-specific CD4+ T cell responses were also associated with higher frequencies of activated CD8+ T cells (n = 28, P = 0.46, P = 0.014, Figure 2B). However, there was no evidence for a relationship between Pol-specific or Nef-specific CD4+ T cell responses and the frequency of activated CD4+ or CD8+ T cells. HIV controllers with higher plasma HIV-specific antibody levels (as assessed by de-tuned ELISA) also had higher frequencies of activated CD4+ T cells (rho: 0.46, P = 0.025) and CD8+ T cells (rho: 0.60, P = 0.002, Figure 2D).

The frequency of HIV-specific CD8+ T cells were less consistently associated with the frequency of activated T cells. In general, there was little evidence for an association between the frequency of HIV-specific CD8+ T cells producing both IFN-γ and IL-2 and the frequency of activated CD4+ or CD8+ T cells. However, higher frequencies of activated CD8+ T cells tended to be associated with higher frequencies of CD8+ T cells producing IFN-γ but not IL-2 in response to HIV Nef (rho: 0.42, P = 0.025, Figure 2C), Pol (rho: 0.39, P = 0.045), and Gag peptides (rho:0.22, P = 0.14).

### HIV controllers also have high CMV-specific CD4+ T cell responses

**Figure 3:**
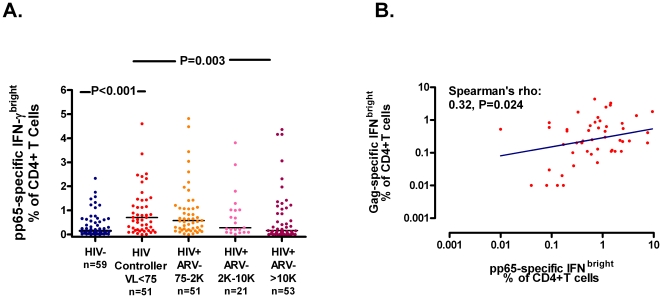
HIV Controllers Have High Frequencies of CMV-specific T Cells. (**A**) The frequency of CD4+ T cells producing IFN-γ after incubation with CMV pp65 peptides *in vitro* (representative flow plot depicted in Figure 1 from reference [85]) was assessed in HIV-uninfected but CMV-seropositive controls, HIV controllers, and untreated HIV-infected participants with varying degrees of detectable viremia (75–2000, 2001–10,000, and >10,000 copies/ml). The HIV controllers had higher CMV pp65-specific IFN-bright CD4+ T cell responses than HIV-uninfected controls and HIV-infected participants with high levels of viremia. HIV controllers with higher CMV pp65-specific CD4+ T cell responses also had higher HIV Gag-specific CD4+ T cell responses (curve represents linear regression model on untransformed data, **B**).

We next hypothesized that an unusually low Treg response in HIV controllers might also contribute to higher adaptive immune responses directed at other chronic viral infections. We chose to focus on cytomegalovirus (CMV) since CMV is nearly ubiquitous in HIV infected individuals, is typically controlled to nearly undetectable levels in individuals with intact immune systems, yet elicits high frequencies of CMV-specific T cells even in HIV-uninfected individuals [37], [38]. To address this, we compared CMV-specific T cell responses between HIV-uninfected but CMV-sero-positive controls, HIV controllers, and untreated HIV-infected participants with varying plasma HIV RNA levels (75–2000, 2001–10,000, and >10,000 copies/ml). The HIV controllers had higher CMV pp65-specific IFN-bright CD4+ T cell responses than HIV-uninfected controls (P<0.001, Figure 3A). While HIV controllers had similar frequencies of pp65-specific IFN-bright CD4+ T cells as untreated HIV-infected participants maintaining low but detectable plasma HIV RNA levels between 75 and 2,000 copies/ml, they had significantly higher frequencies of pp65-specific IFN-bright CD4+ T cells than HIV-infected participants with plasma HIV RNA levels >10,000 copies/ml (P = 0.003). Across all 4 groups of untreated HIV-infected participants, lower plasma HIV RNA levels were associated with higher pp65-specific CD4+ T cell frequencies (P = 0.001). Even after adjustment for age, HIV controllers continued to have higher pp65-specific CD4+ T cell responses than HIV-uninfected participants (P = 0.003) and untreated HIV-infected participants with plasma HIV RNA levels >10,000 copies/ml (P = 0.016). Notably, HIV controllers with the highest frequencies of pp65-specific CD4+ T cells also had the highest frequencies of Gag-specific CD4+ T cells (rho: 0.32, P = 0.024, Figure 3B). Similar trends were observed when comparing the frequency of CMV-specific IFN-γ+ IL-2+ CD4+ T cells across groups in a smaller subset of individuals (data not shown). There was no evidence for a consistent relationship between pp65-specific CD8+ T cell responses and plasma HIV RNA levels among untreated HIV-infected individuals.

## Discussion

**Figure 4:**
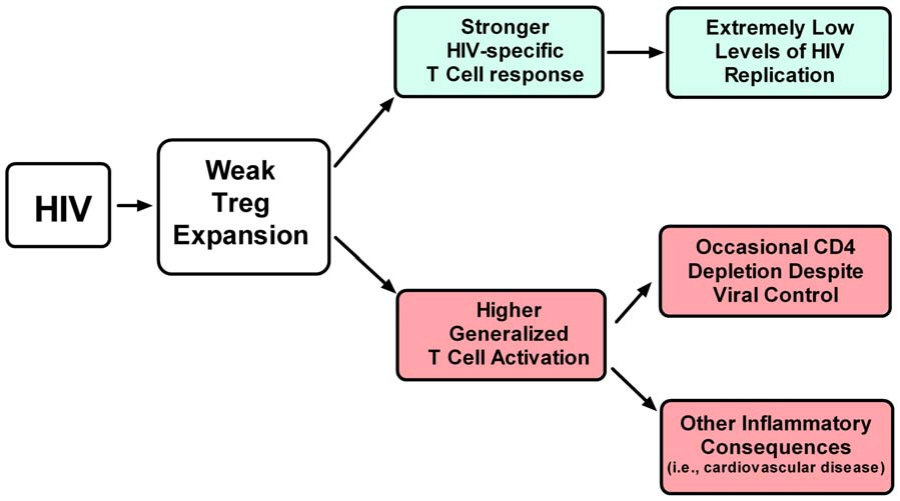
Theoretical Model to Describe Positive and Negative Consequences of Low Treg Frequencies in HIV Controllers. A theoretical model to describe the potential positive and negative consequences of low Treg frequencies in HIV controllers is presented. While a low Treg response might increase HIV-specific T cell responses, contributing to the clearance of HIV-infected cells and the maintenance of extremely low levels of viral replication, a low Treg response might also increase generalized T cell activation, contributing to CD4+ T cell decline and other inflammation-associated comorbidities even in the presence of very low levels of viral replication.

A wealth of data now suggest that most HIV controllers maintain control of viral replication at least in part through potent HIV-specific T cell responses [6], [7], [8], [9], [10], [11], [12], [13], [14], [20], [22], observations that have spurred the development of vaccines that elicit T cell responses against HIV. However, the mechanisms responsible for a strong HIV-specific T cell response in HIV controllers may not be without important consequences for the immune system. As our group recently reported, most HIV controllers have abnormally high levels of immune activation, which is associated with significant CD4+ T cell depletion and even AIDS despite continued control of virus replication [34]. In the current study, we have expanded upon this prior work and assessed potential mechanisms to explain this paradox. First, despite abnormally high T cell activation levels, HIV controllers have significantly lower Treg frequencies than HIV-uninfected individuals. Second, we observed a strikingly strong relationship between adaptive HIV-specific CD4+ T cell and antibody responses and generalized T cell activation in HIV controllers. Third, we observed unusually high CMV-specific CD4+ T cell responses in HIV controllers, suggesting that their ability to mount strong T cell responses to chronic viral infections may not be specific for HIV. Collectively, these observations suggest that a low Treg response may allow some HIV controllers to maintain viral control with a strong cytotoxic HIV-specific T cell response, but might also contribute to the negative inflammatory consequences of generalized T cell activation in this setting (Figure 4).

Multiple mechanisms have been proposed to explain why HIV controllers maintain low to undetectable levels of viral replication in the absence of therapy. While it is possible that some HIV controllers may simply be infected with defective viruses [39], most harbor replication competent viruses that lack gross deletions or lethal mutations [40], [41]. Several lines of evidence suggest an important role of HIV-specific T cells in the control of viral replication. For example, most HIV controllers maintain unusually high frequencies of HIV-specific CD4+ and CD8+ T cells [6], [7], , as well as HIV-specific CD8+ T cells with greater proliferative and cytotoxic potential [8], [12], [42]. While strong HIV-specific T cell responses could conceivably be a consequence of poor viral fitness [43], [44], HIV controllers are highly enriched for protective class I HLA alleles (i.e., B5701) and polymorphisms associated with HLA C expression [15], [16], [17], [18], suggesting that CD8+ T cell responses may play an important role in the control of HIV replication. Some HIV controllers also have high frequencies of CD4+ T cells with cytotoxic activity [45], [46]. However, many HIV controllers lack a protective HLA type, have very low frequencies of HIV-specific T cells, or maintain control of viral replication even after documented escape from HLA-restricted epitopes [14], [20], [21], [22]. In these individuals, non-T cell-mediated mechanisms of control are likely. For example, HIV controllers are highly enriched for HLA and KIR allotypes associated with enhanced natural killer cell responses [47], [48]. Other immunologic mechanisms and host restriction factors that are yet to be fully characterized are also likely to play a role [16], [49].

It is important to acknowledge this heterogeneity in the mechanisms of viral control in HIV controllers as some mechanisms are likely to be associated with more negative inflammatory consequences than others. While other cohorts have not observed increased T cell activation levels in HIV controllers [50], [51], these studies either included individuals with nef-deleted viruses or only included HIV controllers maintaining normal CD4+ T cell counts. When selecting HIV controllers solely on the basis of their ability to control viral replication, it is clear that some controllers eventually progress to significant levels of CD4+ T cell depletion [5], [52], [53], and these individuals have the highest T cell activation levels [34]. In the current study, we also observed that HIV controllers with the highest HIV-specific CD4+ T cell frequencies and antibody levels had the highest levels of generalized T cell activation and the greatest degree of CD4+ T cell depletion. Thus, the HIV-specific immune response and generalized T cell activation are tightly linked in HIV controllers and these relationships appear to be stronger than those observed in untreated HIV-infected individuals with high levels of viral replication [54], [55]. While we cannot exclude the possibility that higher adaptive immune responses are simply a consequence of greater degrees of low-level viral replication - particularly in lymphoid tissues, differences between HIV controllers in the degree of adaptive immune responses and T cell activation may well reflect host differences in the immune response elicited by any given level of virus replication. The extent of microbial translocation may be one factor modulating the response to low-level HIV replication. As we reported previously, most HIV controllers have abnormally high plasma lipopolysaccharide levels [34], which might drive generalized immune activation, but also serve as an adjuvant for HIV-specific T cell responses, particularly in gut-associated lymphoid tissue where the majority of HIV replication is thought to occur.

Alternatively, HIV controllers may be enriched for host genetic factors associated with strong innate and/or weak Treg responses to viral infection. Indeed, we found that HIV controllers had significantly lower frequencies of CD25+CD127^dim^ CD4+ Tregs in peripheral blood than HIV-uninfected individuals despite much higher levels of T cell activation. While we cannot exclude the possibility that HIV controllers preferentially retain Tregs in lymphoid tissues, a recent study also found low frequencies of Tregs in tissues of HIV controllers [56]. While the specific mechanisms mediating the unusually low Treg frequencies in HIV controllers remain unclear, a low Treg response is likely to have competing effects in this setting. For example, several studies have argued that that these cells are detrimental in HIV infection by inhibiting HIV-specific T cell responses [56], [57], [58], [59], [60], [61], while others have argued that these cells are beneficial by reducing generalized T cell activation [62], [63], [64], [65]. Inferring causal relationships is particularly challenging in cross-sectional studies of *in vivo* Treg frequency in HIV-infected individuals since Tregs may be induced and expanded by viral replication and resultant inflammation [36], but once induced, act to decrease inflammation. Accordingly, we observed that HIV controllers with higher levels of immune activation had higher frequencies of Tregs, suggesting that inflammation was driving the induction of Tregs. However, HIV controllers had lower Treg frequencies than HIV-uninfected individuals despite having much higher T cell activation, suggesting a strikingly low Treg response for the degree of immune activation observed. This unusually low Treg response in HIV controllers is therefore likely to be a significant contributor to the high generalized T cell activation and HIV-specific T cell responses observed. These results are consistent with a recent report of decreased inhibitory immunoregulatory receptor CTLA-4 expression on CD4+ T cells in HIV controllers [66].

Our results differ from another recent report describing preserved Treg frequencies (as defined by FoxP3 expression) in the peripheral blood of a much smaller cohort of 12 HIV controllers [50]. However, FoxP3 can be expressed early in the activation of effector CD4+ T cells without any regulatory function [67], [68], [69], [70], [71], [72], so the preserved FoxP3 expression described in that study may simply reflect the presence of recently activated effector CD4+ T cells, particularly since the co-expression of CD25 and FoxP3 in CD4+ T cells was not presented. Low expression of CD127, as measured in our study, may help distinguish Tregs from activated T cells and is now routinely used with CD25 to quantify the frequency of Tregs with suppressor function [73], [74], [75]. It should be noted that among HIV-infected individuals with high levels of viral replication, gating on CD4+/CD25+/CD127^dim^ may include some cells that do not express FoxP3 and thereby lack regulatory function [76]. However, Treg frequencies defined by CD4+/CD25+/CD127^dim^ and CD4+/CD25^hi^/FoxP3+ are highly correlated in HIV-infected individuals with undetectable plasma HIV RNA levels (r = 0.91, P<0.001) [76]. Thus, the low frequency of CD4+/CD25+/CD127^dim^ cells we observed in HIV controllers relative to HIV-uninfected controls and ART-suppressed individuals (all groups with undetectable viremia) almost certainly reflects a low frequency of Tregs in HIV controllers. Lastly, even if HIV controllers had similar levels of Tregs to HIV-uninfected individuals as has been suggested in another recent report using HIV controller samples from the same cohort [77], they would still have unusually low Treg frequencies relative to the expansion of activated T cells observed.

Consistent with the hypothesis that HIV controllers are predisposed to a weak Treg response to chronic viral infections, we observed significantly higher CMV-specific CD4+ T cell responses in HIV controllers than non-controllers and HIV-uninfected individuals. While we cannot exclude the possibility that greater CMV shedding explains the higher CMV-specific CD4+ T cell responses in HIV controllers, CMV shedding tends to be lower in individuals with higher CD4+ T cell counts and lower plasma HIV RNA levels [78]. Thus, the expansion of CMV-specific CD4+ T cells in HIV controllers is unlikely to be driven by higher levels of antigen and is more likely to reflect a more robust proliferation of CD4+ T cells in response to CMV infection. HIV controllers co-infected with hepatitis C virus (HCV) might also exhibit stronger HCV-specific responses than individuals with higher levels of HIV replication [79]. While lower levels of HIV replication may allow for preservation of antigen-specific immune responses, the high CMV-specific CD4+ T cell frequency in HIV controllers relative to HIV-uninfected CMV-seropositive individuals cannot be explained by this mechanism alone. While another recent report suggested that HLA B5701+ elite controllers maintain similar CMV- and HCV-specific CD8+ T cell responses as non-controllers, CD4+ T cell responses were not assessed in that study [80], and epidemiologic data suggest that HIV controllers are much more likely to spontaneously clear HCV than viremic HIV-infected individuals and HIV-uninfected individuals infected with HCV [81].

In summary, we have observed that while most elite controllers maintain high HIV-specific T cell responses, most also have abnormally high generalized T cell activation levels, which may occasionally contribute to significant CD4 depletion even in the absence of clinically detectable viremia. Furthermore, those with the highest HIV-specific T cell responses have the highest levels of generalized immune activation, suggesting possible inflammatory consequences of T cell-mediated control of HIV replication. An unusually low regulatory T cell response to HIV infection may well explain this phenomenon. Perhaps the best immune response to HIV infection is one that maintains control of viral replication while minimizing negative inflammatory consequences. Some elite controllers are able to maintain this balance and understanding the mechanisms of control in these individuals is likely to have important implications for HIV vaccine research.

## Materials and Methods

### Participants

#### For comparison of HIV-specific immune responses and T cell activation levels

HIV-infected adults were sampled from the Study of the Consequences of the Protease Inhibitor Era (SCOPE), a clinic-based cohort of over 1000 chronically HIV-infected individuals at the University of California San Francisco. From this cohort, we evaluated three distinct groups of HIV-infected individuals: (1) HIV controllers, defined as HIV-seropositive individuals maintaining plasma HIV RNA levels <75 copies/ml in the absence of therapy (episodes of clinically detectable viremia in the previous year were allowed if they were followed by undetectable values); (2) “ART-suppressed” individuals maintaining plasma HIV RNA levels <75 copies/ml on antiretroviral therapy; and (3) untreated HIV “non-controllers” with plasma HIV RNA levels above 10,000 copies/mL. T cell activation data have been previously reported on 30 of the 52 HIV controllers and all of the ART-suppressed and untreated patients in the current report [34], HIV-specific T cell response data have also been reported on these individuals recently [14]. HIV-uninfected individuals were also sampled from a study of the immunologic determinants of atherosclerosis and have been reported on previously [14], [82].

#### For comparisons of CMV-specific T cell responses between groups

In addition to the above participants, untreated HIV-infected participants with plasma HIV RNA levels between 75 and 10,000 copies/ml were sampled from the SCOPE cohort. HIV-negative individuals were also sampled from a trial of post-exposure prophylaxis following a non-occupational exposure to HIV [83]. Only CMV-seropositive HIV-negative participants were included in the analyses of CMV-specific T cell responses.

#### For comparison of Tregs between groups

Given limited PBMC availability, cryopreserved PBMC from different SCOPE participant-timepoints were sampled for the measurement of both Treg frequency and T cell activation levels in 20 HIV controllers, 20 HAART-suppressed participants, and 20 non-controllers. Only specimens on participants with CD4+ T cell counts >350 cells/mm^3^ were selected for these analyses to ensure adequate overlap between groups. For the Treg analyses, cryopreserved PBMC were also sampled from 34 healthy HIV-uninfected controls from the AIDS Clinical Trials Group 5015 study [84].

### Ethics Statement

All participants provided written informed consent and this research was approved by the institutional review board of the University of California, San Francisco.

### Laboratory Studies

#### T cell activation

Freshly collected, EDTA-anticoagulated whole blood was analyzed by four-color flow cytometry on a Beckman Coulter Epics XL flow cytometer. Blood was stained on a Beckman Coulter Prep Plus and lysed on a Beckman Coulter TQ Prep. Activated (CD38+/HLA-DR+) T cells were identified with FITC-conjugated anti-HLA-DR, PE-conjugated anti-CD38 (both from BD Bioscience), PC5-conjugated anti-CD3 and PE-texas red conjugated anti-CD4 or CD8 (Beckman Coulter). The activation markers CD38 and HLA-DR were gated from the CD3+CD4+ or CD3+CD8+ cells on a 2-dimensional dot plot where quadrant gates, set on an isotype control, were used to define positive and negative populations. T cell activation levels were reported as the percentage of CD4+ and CD8+ T cells expressing both HLA-DR and CD38.

#### Cytokine flow cytometry

Fresh whole blood was stimulated with overlapping peptide pools (15-amino-acid peptides overlapping by 11 amino acids) of the HIV-1 p55 Gag, Pol, Nef, Env, or CMV pp65 protein (BD Biosciences, San Jose, CA) for 6 h in the presence of brefeldin A, as reported recently [14]. Unstimulated cells and superantigen staphylococcal enterotoxin B (Sigma Aldrich)-stimulated cells were used as negative and positive controls, respectively. Cells were fixed, permeabilized, and stained with FITC- conjugated anti-interferon (IFN)-γ, PE-conjugated anti-IL-2, APC-conjugated anti-CD3 (all BD Bioscience) and PC5-conjugated anti-CD4 (Beckman Coulter) and data was collected on a Becton Dickinson FACSCalibur. The fractions of CD4+ and CD8+ T cells secreting IFN-γ and/or IL-2 were determined using FlowJo software (TreeStar). In our primary analysis of CMV-specific T cell responses, we focused on cells that stained brightly for IFN-γ. The “IFN-γ bright” gate was set 3 decades above the IFN-γ-negative population in non-stimulated control, as previously described (representative flow plot depicted in Figure 1 from reference [85]). Cells were initially defined as lymphocytes based on forward- and side-scatter profiles. CD4+ and CD8+ anchor gates were drawn on the CD3+CD4+ and CD3+CD4- populations, respectively. At least 10,000 CD3+CD4+ and CD3+CD4- events were collected for the majority of subjects; data were excluded if <4,000 events were collected. Cytokine secretion levels in the negative control were subtracted to correct for nonspecific cytokine secretion.

#### Treg frequencies

Cryropreserved PBMC were evaluated using 4-color flow cytometry. Mouse anti-human monoclonal antibodies (CD4, CD8, CD25, CD45RO, and CD127) conjugated to fluorescein isothiocyanate (FITC), phycoerythrin (PE), PerCP, and allophycocyanin (APC) from BD Biosciences (San Jose, CA) or Coulter Immunology (Miami, FL) were used to stain the PBMC preparations. Non-specific antibody binding to Fc receptors was blocked by pre-incubation of the cells with Fcγ-receptor block (Miltenyi Biotec, Auburn, CA). All samples were evaluated within 24-hours of staining using a FACSCalibur™ flow cytometer. Logical gating was used to identify the frequency of T regulatory (CD4+/CD25+/CD127^dim^) T lymphocyte populations (Figure 1B) [73], [74], [75]. Results are expressed as the percentage of the parent CD4+ T cell population.

#### HIV Antibody Levels

A “de-tuned” enzyme immunoassay (Organon Tecnika Vironostika [OTV], BioMerieux) was used to measure semiquantitative HIV antibody levels on a subset of HIV controllers [86]. The OTV is a second-generation ELISA that detects both IgG and IgM antibodies to HIV-1 and is FDA-approved for diagnostic testing. The less sensitive modification involves testing 1∶20,000 dilutions of plasma under abbreviated incubation conditions and calculating a standardized optical density (SOD) for each sample [87].

#### Statistical Methods

Continuous variables were compared between groups with Kruskal Wallis tests followed by Wilcoxon ranksum tests for pairwise comparisons. Dichotomous variables were compared between groups with chi square and Fisher's exact tests. Relationships between continuous variables were assessed with Spearman's rank order correlation coefficients. Adjusted differences between groups were assessed with linear regression, calculating standard errors with heteroskedasticity-consistent covariance matrix estimators and log-transforming outcomes when necessary to satisfy model assumptions [88].

## Supporting Information

Figure S1 (TIF)
